# MOBILE RIGHT HEART THROMBUS WITH PULMONARY EMBOLISM IN A PATIENT WITH POLYCYTHEMIA RUBRA VERA AND SPLANCHNIC VEIN THROMBOSIS

**Published:** 2010

**Authors:** Prashanth Panduranga, Mohammed Mukhaini, Muhammad Saleem, Taha Al-Delamie, Sunny Zachariah, Saqar Al-Taie

**Affiliations:** 1 *Department of Cardiology, Royal Hospital, Muscat, Oman*; 2 *Department of Cardiothoracic surgery, Royal Hospital, Muscat, Oman*; 3 *Department of Radiology, Royal Hospital, Muscat, Oman*

**Keywords:** right heart thrombus, Right atrial thrombus, Pulmonary embolism, Polycythemia rubra vera, Splanchnic vein thrombosis

## Abstract

Splanchnic vein thrombosis in patients with polycythemia rubra vera is well-known. Development of mobile right heart thrombus in these patients has not been reported previously. We describe a young patient with Polycythemia rubra vera and splanchnic vein thrombosis with ischemic bowel who underwent small bowel resection. He developed a large mobile right atrial thrombus and bilateral pulmonary embolism. He also had upper gastrointestinal bleed. His management was complicated and challenging due to multiple risk factors and co-morbid conditions. Thrombolysis was contraindicated and he refused surgical intervention. He was treated with anticoagulation with complete resolution of right atrial thrombus.

## Case presentation

A 31-year-old Arab male presented with two days history of diffuse colicky abdominal pain along with guarding and spleno-megaly. He was known to suffer from Polycythemia rubra vera (PRV) for the last eight years and was Janus kinase 2 (JAK2) mutation negative. He was on prophylactic aspirin and regular phlebotomy every 2-3 months.

Laboratory studies showed hemoglobin of 14g/dL, hematocrit of 49% with normal white cell and platelet counts. A computed tomography scan of the abdomen showed extensive thrombosis of superior mesenteric vein (Figure 1A) along with splenic and portal veins (Figure 1B) with evidence of small bowel ischemia.

**Fig 1A:**
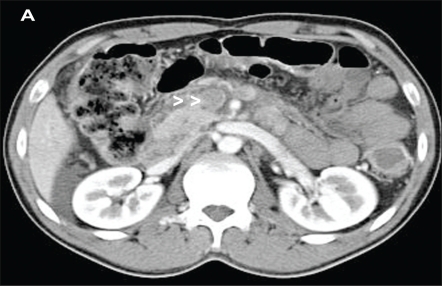
Contrast-enhanced axial computed tomography scan shows clot in superior mesenteric vein (A, arrow heads), portal vein (B, right arrow heads) and splenic veins (C, left arrow heads) in a patient with polycythemia rubra vera and mobile right heart thrombus.

**Fig 1B:**
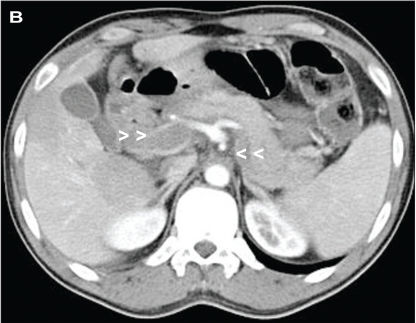
Contrast-enhanced axial computed tomography scan shows portal vein (B, right arrow heads) and splenic veins (C, left arrow heads) in a patient with polycythemia rubra vera and mobile right heart thrombus.

**Fig 2:**
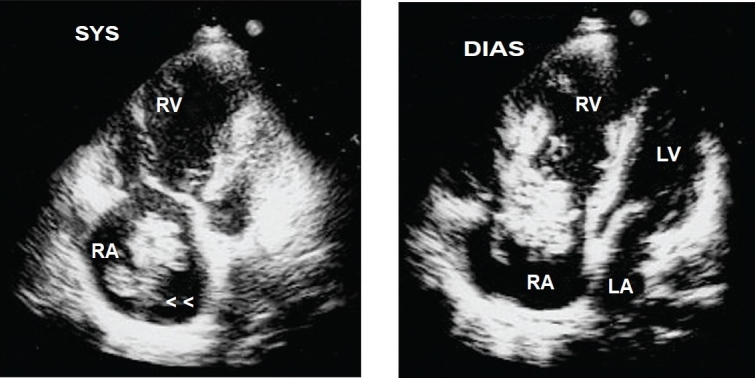
Transthoracic echocardiogram showing a large free-floating thrombus (arrowhead) in the right atrium prolapsing into right ventricle through the tricuspid valve in a patient with polycythemia rubra vera and splanchnic vein thrombosis. **Abbreviations: RA**=Right atrium; **RV**=Right ventricle; **LA**=Left atrium; **LV**=Left ventricle.

Heparin infusion was started. He underwent emergency small bowel resection with end to end anastomosis. On the second post operative day, he developed intermittent narrow QRS atrial tachycardia and became hypoxic and hypotensive with new right bundle branch block on ECG. Transthoracic echocardiography (TTE) showed right atrial (RA) and ventricular dilatation with a large mobile serpigenous free-floating thrombus in the RA (measuring 6.0cm × 3.0cm) and prolapsing into the right ventricle (RV) through the tricuspid valve leaflets (Figure 2). There was severe tricuspid regurgitation with RV systolic pressure of 70 mmHg. Left ventricle was small with good systolic function.

Computed tomography pulmonary angiogram showed bilateral right and left pulmonary artery emboli (Figure 3A and B). Ultrasound Doppler of inferior vena cava and lower limb veins did not show any thrombi. He also developed intermittent melena and gastroscopy showed Grade I esophageal varices. He was stabilized with blood transfusion and inotropes. He was advised surgical removal of the RA thrombus but he declined. Thrombolysis was contraindicated and hence, he was continued on heparin with overlap warfarin. He improved hemodynamically with no recurrence of atrial tachycardia. A repeat TTE after two weeks showed complete resolution of RA thrombus (Figure 4) with RV systolic pressure of 45mmHg.

**Fig 3A:**
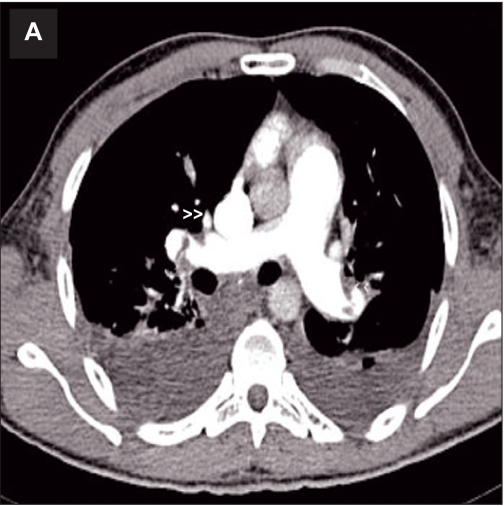
Computed tomography pulmonary angiogram showing right and left pulmonary artery emboli (A, arrow heads) and extensive thrombus in right pulmonary artery (B, arrow) in a patient with polycythemia rubra vera, splanchnic vein thrombosis and mobile right heart thrombus.

**Fig 3B:**
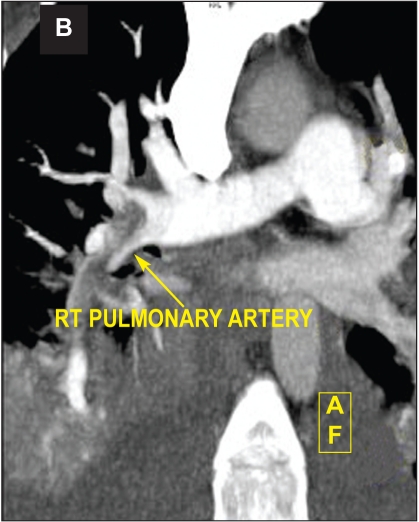
Computed tomography pulmonary angiogram showing (B, arrow) in a patient with polycythemia rubra vera, splanchnic vein thrombosis and mobile right heart thrombus.

**Fig 4:**
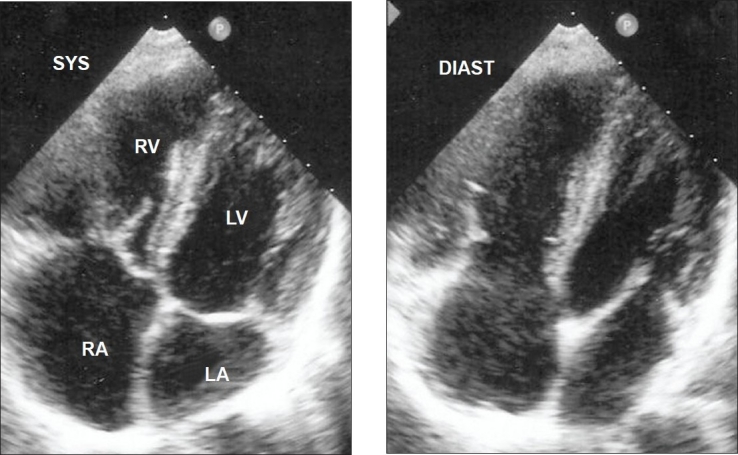
Transthoracic echocardiogram showing complete resolution of right atrial thrombus with oral anticoagulation in a patient with polycythemia rubra vera, splanchnic vein thrombosis and mobile right heart thrombus. Abbreviations as before.

## Discussion

PRV is a chronic myeloproliferative disease associated with an increased risk of venous or arterial thrombosis and hemorrhage. Phlebotomy, cytoreductive treatment, and prophylactic aspirin have reduced the number of thrombotic complications^1^. In patients with PRV, splanchnic vein thrombosis is often an early complication and can manifest before diagnosis. Increased whole blood viscosity is the most likely cause for thrombotic events in patients with PRV. However, splanchnic vein thrombosis can occur in patients with PRV with normal hematocrit, blood cell and platelet levels (as in our patient) either due to concomitant portal hypertension and hypersplenism which may mask any increase in blood cell counts or due to cytoreductive therapy and phlebotomy. Recently, JAK2 gene mutation was described in patients with splanchnic vein thrombosis and PRV^2^ but the results from many studies are conflicting. Our patient was JAK2 negative.

The interesting event in our patient was development of a large mobile right heart thrombus (MRHT) which has not been described before in a patient with PRV and splanchnic vein thrombosis. Right heart thrombi are commonly described in patients with atrial fibrillation/flutter, prolonged central venous catheters, or transvenous pacing leads. Primary thrombi develop within the RA (in-situ thrombi) and are usually immobile attached to atrial wall. Secondary thrombi result from venous embolization and are temporarily lodged in the right atrium or ventricle (emboli in transit) and are usually highly mobile (MRHT). MRHT are a rare phenomenon, generally diagnosed when echocardiography is performed in patients with suspected or proven pulmonary embolism (PE)^3^. MRHT have been variously described as spherical, coiled, ovoid, worm like or serpiginous masses moving within the right atrium or ventricle prolapsing into the tricuspid or pulmonic valve. They are free-floating with no point of attachment seen. The serpigenous nature of the migrating thromboemboli is characteristic and, in contrast, a pedunculated mass attached to the interatrial septum suggests the diagnosis of myxoma. In the case presented, the thrombus in RA was related to the thrombus in the splanchnic veins which was demonstrated by abdominal computed tomography.

Earlier smaller studies have reported an echocardiographic prevalence of MRHT from 7% to 18% among patients with PE^3^,^4^,^5^. In a large autopsy study, all the patients with PE at autopsy, right heart thrombus was found in 7%, and the only detected source of PE in 4.0%^6^. Analysis of a European multicentre PE registry involving 1,135 patients with PE showed echocardiographic prevalence of MRHT to be around 4%^7^. Patients with MRHT were more haemodynamically compromised, had more frequent RV hypokinesis along with double the frequency of clinical congestive heart failure and more often had right bundle branch block on electrocardiogram. All these features were present in our patient.

TTE is the first investigation to detect MRHT complimented by transesophageal echocardio-graphy with^8^ or without contrast and /or cine magnetic resonance imaging^9^. Some authors have the opinion that TTE may underestimate the true prevalence of MRHT due to low sensitivity in detecting right heart thrombi^10^. Hence, it is recommended that transesophageal echocardiography be done in cases where TTE is not diagnostic in patients with PE especially when no source is detected.

MRHT can embolize at any moment, either before or after thrombolysis. Treatment for MRHT includes surgical embolectomy, intravenous thrombolysis, and intravenous heparin. In a large meta-analysis of MRHT studies, the overall mortality was 27% and mortality post thrombolysis was significantly lower than with surgery or anticoagulation alone (11.3% vs 23.8% and 28.6% respectively)^10^. Analysis of a European multicentre PE registry showed no overall difference in mortality between the three treatment modalities. However, subgroup analysis showed increased mortality in comparable groups of patients with and without MRHT treated with heparin alone (23.5% vs 8% respectively)^7^. In one study, there was 50% rate of MRHT disappearance after two hours of thrombolysis^11^. Furthermore, thrombolytic agents are known to reverse RV dysfunction early in patients with major PE^12^. These findings suggest that anticoagulation alone may not be sufficient treatment for patients with MRHT and that thrombolysis is the preferred treatment if no contraindications are present followed by surgical embolectomy.

The overall risk of hemorrhage with thrombolysis is reported as 6-20%, with no significant differences between the alternative agents^13^. Our patient was not treated with thrombolytic therapy because of recent laparotomy and upper gastrointestinal bleed. He denied surgical intervention. Interventional techniques using basket device to trap the thrombus into the inferior vena cava with placement of caval filter above the thrombus has been described previously but we do not have experience with this procedure^3^. However, our patient responded very well to anticoagulation with complete resolution of RA thrombus as well as decrease in pulmonary artery pressures.

In conclusion, this case report presents a patient with very rare combination of polycythemia rubra vera with splanchnic vein thrombosis and mobile right heart thrombi along with pulmonary embolism who was successfully managed with anticoagulation. In these patients, the choice of anticoagulation, thrombolysis, or surgery will depend on additional risk factors and co-morbid conditions.

## References

[1] Landolfi R, Marchioli R, Kutti J, Gisslinger H, Tognoni G, Patrono C (2004). Efficacy and Safety of low-dose aspirin in polycythemia vera: Results of the European Collaboration on Low-dose Aspirin in Polycythemia Vera (ECLAP) trial. N Engl J Med.

[2] Colaizzo D, Amitrano L, Tiscia GL, Grandone E, Guardascione MA, Margaglione M (2007). A new JAK2 gene mutation in patients with polycythemia vera and splanchnic vein thrombosis. Blood.

[3] Chartier L, Bera J, Delomez M, Asseman P, Beregi J, Bauchart JJ (1999). Free-floating thrombi in the right heart: diagnosis, management, and prognostic indexes in 38 consecutive patients. Circulation.

[4] Chapoutot L, Nazeyrollas P, Metz D, Maes D, Maillier B, Jennesseaux C (1996). Floating right heart thrombi and pulmonary embolism: diagnosis, outcome and therapeutic management. *Cardiology*.

[5] Casazza F, Bongarzoni A, Centonze F, Morpurgo M (1997). Prevalence and prognostic significance of right-sided cardiac mobile thrombi in acute massive pulmonary embolism. *Am J Cardiol*.

[6] Ogren M, Bergqvist D, Eriksson H, Lindblad B, Sternby NH (2005). Prevalence and risk of pulmonary embolism in patients with intracardiac thrombosis: a population-based study of 23,796 consecutive autopsies. *Eur Heart J*.

[7] Torbiki A, Galie N, Covezzoli A, Rossi E, Goldhaber S (2003). Right heart thrombi in pulmonary embolism: results from the international cooperative pulmonary embolism registry. *J Am Coll Cardiol*.

[8] Calleja ACM, Alharthi MS, Khandheria BK, Chaliki HP (2009). Contrast-enhanced right atrial mass: tumour or thrombus?. *Eur J Echocardiogr*.

[9] X Hou, W Liu, Z Zhang, Z Li (2009). Free-floating right atrial thrombus with acute pulmonary embolism. *Thorax*.

[10] Rose PS, Punjabi NM, Pearse DB (2002). Treatment of Right Heart Thromboemboli. *Chest*.

[11] Ferrari E, Benhamou M, Berthier F, Baudouy M (2005). Mobile Thrombi of the Right Heart in pulmonary Embolism- Delayed Disappearance After Thrombolytic Treatment. *Chest*.

[12] Zhu L, Wang C, Yang Y, Wu Y, Zhai Z, Dai H et al (2007). Value of transthoracic echocardiography in therapy regimens evaluation in pulmonary embolism. *J Thromb Thrombolysis*.

[13] Harris T, Meek S (2005). When should we thrombolyse patients with pulmonary embolism. A systematic review of the literature?. *Emerg Med*.

